# Chronic *Toxoplasma gondii* infection and its influence on semen analysis in rats: controlled experimental animal study

**DOI:** 10.1038/s41598-026-62773-z

**Published:** 2026-08-24

**Authors:** Samar Nagah El-Beshbishi, Soha Ibrahim Awad, Elham Farag Almeniar, Mona Younis Youssef, Nora El-Tantawy, Amira Ibrahim Taman

**Affiliations:** 1 Department of Medical Parasitology, Faculty of Medicine, Mansoura University, Mansoura, Egypt; 2 Department of Basic Medical Science, Faculty of Medicine, New Mansoura University, New Mansoura, Egypt; 3 Department of Anatomic Pathology, Faculty of Medicine, Mansoura University, Mansoura, Egypt; 4 Department of Public Health, Faculty of Applied Medical Sciences, Al-Baha University, Al- Baha, Saudi Arabia; 5 Department of Parasitology, Program of Medicine and Surgery, Faculty of Medicine, Mansoura National University, Gamasa, Egypt

**Keywords:** *Toxoplasma gondii*, Male, Experimental, Rat, Sperm, Infertility, Diseases, Microbiology

## Abstract

Infertility is a significant global health concern that has been related to various infectious agents. While *Toxoplasma gondii* (*T. gondii*) is a ubiquitous protozoan parasite that affects various body organs, little is known concerning how it may affect the male reproductive system. This research aims to study the impact of *T. gondii* infection on semen analysis in experimentally infected rats, focusing on the parameters of sperm count, motility, and morphological integrity. This controlled experimental animal study involved 36 outbred male Wistar rats (24 cases and 12 controls). The rats were infected with the ME-49 strain of *T. gondii*. The rats were euthanized at days 40, 50, and 60 PI, and the testes and epididymides were removed immediately. The body weight testis weight ratio was assessed. Chronic toxoplasmosis was evaluated by examination of the brain for *T. gondii* cysts. Histopathological examination of the brain and scoring of its lesions were performed. Semen analysis was performed, and sperm parameters were assessed for count, motility, viability and morphological characteristics. The correlations between the sperm parameters and the brain inflammation score and the brain cyst burden were investigated. Cysts of *Toxoplasma* were found in the brain homogenates of all infected rats. The median percentage of progressively motile sperm, median sperm count, and median percentage of viable sperm in the *T. gondii*-infected group were significantly lower than those in the control group (*P*<.05). At all-time points, the percentage of normal sperm in the infected rats was significantly lower than that in the control rats. At all-time points, the *T. gondii* infected rat group had a considerably greater median proportion of morphologically abnormal sperm than did the control group (*P*<.05). This study demonstrated that *T. gondii* infection significantly impacts semen quality in an experimental rat model. Chronic toxoplasmosis is associated with significant reductions in sperm count, motility, and viability and an increase in morphologically abnormal sperm. The demonstrated alterations in semen parameters highlight the potential role of *T. gondii* as a contributing factor to male rat reduced fertility.

## Introduction

A significant public health issue worldwide, more than 7% of males have infertility; cases of idiopathic male infertility have been documented, with a nearly 50% prevalence^1^. Male causes of infertility include poor sperm motility, low sperm count, and aberrant sperm morphology^2^. One of the most prevalent and widespread parasitic zoonoses worldwide is *Toxoplasma gondii* (*T. gondii*); approximately 25% of the global population is infected^3^. There are several ways that *T. gondii* can spread, including orally, congenitally, by organ transplantation, and occasionally, through blood transfusion. The rapidly replicating tachyzoites in tissue cysts change into slowly dividing bradyzoites, which are characteristic of latent toxoplasmosis, in most immunocompetent individuals after an initial acute phase of the disease. This transition, does not occur in immunocompromised patients, and the illness can progress to encephalitis and death^4^.

Male infertility has been shown to be associated with a higher incidence of acute and chronic toxoplasmosis^5^. Additionally, infertile men had the highest scores for chronic *T. gondii* infections^6^. The elevated anti-sperm antibody levels in couples infected with *T. gondii* may explain why *T. gondii* infection is more common in human couples who are infertile than in those who are fertile^7^. Among 100 male infertile cases, 36% were positive for *Toxoplasma*-IgG, IgM and circulating antigens^8^.

Acute *T. gondii* infection has been implicated in male infertility. Male mice infected with *T. gondii* exhibited pathological changes in their testes, epididymides, vas deferens, and prostate during the acute phase of infection^9^. Acute *T. gondii* infection has also been shown to adversely impact the reproductivity of experimentally infected male mice^10^. Additionally, *T. gondii* has been detected in the reproductive organs and semen of experimentally infected male rats^11,12^. *T. gondii* tissue cysts have been discovered in the brain and pituitary gland without any other abnormalities, although toxoplasmosis negatively affects primary reproductive indices, such as sperm morphology, motility, and concentration, in male rats^13^. Reproductive parameter impairment associated with toxoplasmosis in male rats appears to be transient rather than permanent^14^.

The intracellular protozoan *T. gondii* has been linked to spermatogenesis and male reproductive disorders. Studies have demonstrated that chronic toxoplasmosis interferes with immunological homeostasis and hormonal balance, both of which are essential for healthy sperm production^15^. It has been demonstrated that infection causes inflammation and oxidative stress in the testes, which can harm the germinal epithelium and decrease the quality of sperm^16^. Furthermore, infected individuals have been found to have disturbances in testosterone and other gonadotropic hormones, which further impair spermatogenesis^17^.

Interference with sperm function is one potential explanation for infertility caused by *T. gondii* infection. Studies has shown that male with toxoplasmosis exhibit reduced sperm parameters, including motility, concentration, and viability^13,14,18^. Furthermore, apoptosis, DNA damage to testicles and sperm, alterations in oxygen free radicals, and superoxide dismutase in the testes are related to acute toxoplasmosis^9,13^. The ME-49 strain is a well-characterized low-virulence type II *T. gondii* strain, which can induce a chronic infection in rodents without high mortality rates. This is the most used strain for the long-term effects of chronic toxoplasmosis on host physiology^19^. The aim of this study was to investigate the effects of experimentally induced chronic toxoplasmosis on semen parameters including count, viability, motility, and morphological integrity as key indicators of male fertility potential.

## Methods

### Study design

This controlled experimental animal study was conducted at Mansoura University’s Faculty of Medicine’s Department of Medical Parasitology from January 2022 until February 2023. The study was conducted in accordance with the National Institutes of Laboratory Animal Resources’ “Guide for the Care and Use of Laboratory Animals” published in the United States. For all surgeries, pentobarbital sodium anesthesia was used, and every effort was made to reduce the suffering and number of rats used. The Institutional Review Board of Mansoura University’s Faculty of Medicine in Egypt reviewed and approved the study proposal (MFM-IRB approval number: MD.17.03.153). The research was conducted carefully in accordance with the rules of the Institutional Council on Research Ethics and is reported according to ARRIVE standards. All the experiments were conducted in compliance with the applicable rules and regulations. The sample size was set a priori to accommodate balanced cross-sectional design at three post-infection (PI) time points: 8 infected rats and 4 control rats were sacrificed at each predetermined time point: Day 40 PI, Day 50 PI, and Day 60 PI. This totals 24 infected and 12 control animals in total. This sample size is comparable to that used in other experimental rodent studies on chronic toxoplasmosis and reproductive parameters^14,20^. However, formal power calculations were not performed for all outcome variables which represent a methodological limitation.

### Experimental model of toxoplasmosis

In this study, Wistar male rats were infected with the low-virulence ME-49 strain of *T. gondii*. This strain’s brain cysts were kindly provided by the Faculty of Medicine at Alexandria University. For one-year, Swiss Webster mice received a parasite dose of ten tissue cysts orally via a 22-gauge blunt feeding needle. The Mansoura Experimental Research Center’s (MERC) animal research unit, located in the Faculty of Medicine, Mansoura University, supplied all the mice and rats used for the study.

For the establishment of chronic infection, outbred male and female Swiss Webster mice weighing 25–30 g and aged 6–8 weeks were used. Food and water were freely available to the mice. They were kept in cages containing five to seven mice each (males and females were kept apart). They were housed in a room with a twelve-hour light/dark cycle, 21 ± 2 °C temperature control, and 60–70% relative humidity. For the experiment, thirty-six Wistar outbred male rats weighing 250 to 280 g were used. Two rats were housed per cage under 1 10-12- hour light/dark cycle in an air-conditioned animal room at a temperature of 25 °C ± 2 and 60% ± relative humidity. Ad libitum access to water and standard rodent chow was available. The animals were divided randomly into two groups of rats following one week of acclimatization; twelve rats were used as controls in group 2, whereas twenty-four rats in group 1 were infected with *T. gondii*. One week after acclimatization, all rats were ear-tagged and randomly distributed into different groups for the experiment by a random number generator. A total of twenty-four rats were randomly distributed into the infected group (Group 1), and twelve rats were distributed into the uninfected control group (Group 2). Among the infected group of rats, 8 rats were randomly distributed into each of the three necropsy time points of Day 40, 50, and 60 PI. The same number of rats (4 rats) from the uninfected control group was also randomly distributed into each of the three necropsy time points.

### Preparation of the cyst inoculum and induction of infection

Eight weeks after oral infection, *Toxoplasma*-infected mice received an intraperitoneal injection of pentobarbital sodium (40 mg/kg) for anesthesia. To obtain the tissue cysts needed to conduct the experiment, the mice were then sacrificed by cervical dislocation, and brain tissue was obtained by decapitation. The brain tissues of the infected mice were homogenized via a Teflon homogenizer with 1 mL of phosphate-buffered saline (PBS). A hemocytometer with 40X magnification was used to count the number of cysts in 10 µl of brain homogenate on the basis of^21^. The experiment involved administering 15 tissue cysts in 100 µl of brain homogenate orally to male Wistar rats in group 1 (the infected group) via a 22-gauge blunt feeding needle^22^. Group 2 (controls) received only regular saline.

### Analysis of acute toxoplasmosis clinical signs

Weekly body weight assessments were performed for the infected and control groups. Every week, the posture, activity, and fur of the Wistar rats were assessed via the following scoring method, per^23^: a score of 0 indicates glossy, smooth fur and active rats; a score of hunched posture and dull or ruffled fur of the rats; and a score of II indicates that the rats were reluctant to move.

### Evaluation of chronic toxoplasmosis and the degree of brain tissue infection

At every scheduled point (40, 50 and 60 days PI), 8 rats from the infected group and 4 rats from the control group were anesthetized via inhalation of halothane. Euthanasia was performed by exsanguination. The animals were then subjected to wide opening of the abdominal cavity to expose the reproductive organs, and the testes and epididymis were immediately removed. Decapitation of the brain was performed for further examination. All rats were weighed before infection and immediately before necropsy. The rats were euthanized, and their brains were removed. As Dubey^24^ reported, each rat’s brain was homogenized in 1 mL of PBS^25^. To determine the brain burden, 10 µl of brain homogenate was examined at 100X magnification to count the number of cysts, either unstained or after Giemsa staining^26^. The brain cysts burden was calculated as the number of cysts in 10 µl of homogenate×100 × 2 was the brain cyst burden.

### Histopathological scoring of brain lesions

Following fixation of one cerebral hemisphere in 5% neutral buffered formalin for 48 h, paraffin embedding, sectioning at 5 μm, and staining with hematoxylin and eosin (H&E), two sections per animal were examined under a 40× microscope by a single board-certified anatomic pathologist who was unaware of the study group allocations and post-infection time point. Tanaka et al.^27^ reported that the following scheme was employed to evaluate the score of inflammation (from 0 to IV) in tissues of the brain in 2 sections at 40x magnification: a score of 0 indicates that there is no lesion; a score of I indicates that there is a minimal lesion confined to localized perivascular cuffs with some infiltration of mononuclear cells in the meninges; and a score of II denotes a mild lesion that includes meningitis, perivascular cuffs, and local glial cell infiltration. A score of III represents moderate disease and includes glial cell activation, perivascular cuffs, focal necrosis, meningitis, and neuropil rarefaction with some macrophage infiltration. A lesion with a score of IV is considered severe and includes perivascular cuffs, glial cell activation, meningitis, neuropil rarefaction, and localized extensive necrosis.

### Preparation of the testes and epididymis

In accordance with^16^, male rats that had been briefly necropsied were placed on a necropsy board, and a 5 cm incision was made in the lower right abdominal region to reach the abdominal cavity. Then, a pair of forceps was used to grab the epididymal fat, and the testis and epididymis were extracted, preserving the vas deferens attached for reference. An incision was made approximately 1 mm from the point where the epididymis cauda and vas deferens join to detach the epididymis from the testis and obtain the epididymal cauda.

### Body weight to testis weight ratio

The ratio of body weight to testis weight was determined via the following method, per^14^, after which the mean weight of each rat’s testes was measured and recorded in grams. Prior to necropsy, the final body weight × 100 was divided by the mean weight of both testes.

### Sperm parameter evaluation

The criteria for semen analysis were used to examine sperm parameters such as count, motility, viability, and morphology^28^. Sperm morphological classification was conducted based on coded photomicrographs to exclude observer bias. However, it is acknowledged that blinding of all sperm parameter measurement (motility, count, and viability) could not be fully carried out due to operational constraints, as these measurements were conducted immediately after collection of samples at scheduled necropsy time points.

#### Epididymal sperm motility

At 40x magnification, a 10-microliter wet preparation of fresh semen was placed on a microscope slide and covered with a 22 mm×22 mm coverslip. There were at least two hundred spermatozoa in each replication, which were distributed throughout at least five fields according to the^28^. The sperm motility was determined by counting both motile and non-motile spermatozoa and the average was calculated. The percentage of motile spermatozoa was calculated using this formula^29^: $\:\%\:motility=\frac{\mathrm{T}\mathrm{o}\mathrm{t}\mathrm{a}\mathrm{l}\:\mathrm{s}\mathrm{p}\mathrm{e}\mathrm{r}\mathrm{m}\mathrm{s}\:-\:\mathrm{N}\mathrm{o}\mathrm{n}\:\mathrm{m}\mathrm{o}\mathrm{t}\mathrm{i}\mathrm{l}\mathrm{e}\:\:\mathrm{s}\mathrm{p}\mathrm{e}\mathrm{r}\mathrm{m}\mathrm{s}}{\mathrm{T}\mathrm{o}\mathrm{t}\mathrm{a}\mathrm{l}\:\mathrm{s}\mathrm{p}\mathrm{e}\mathrm{r}\mathrm{m}\mathrm{s}}$×100.

#### Sperm count

**Fig. 1:**
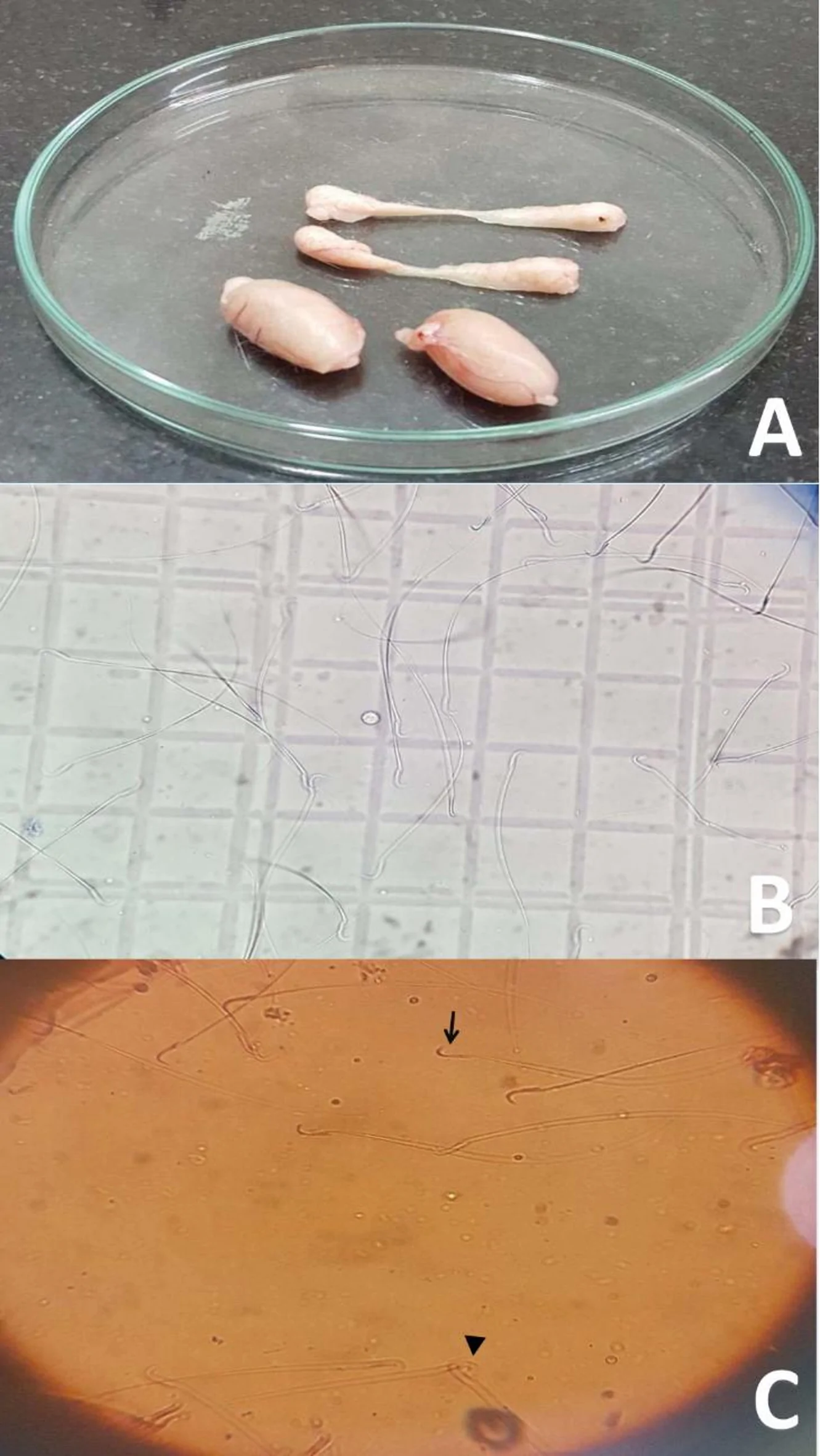
(**A**) Testes and epididymides of male Wistar rats after dissection. (**B**) Rat sperm in the counting area of the hemocytometer (40X magnification). (**C**) Viable (arrowhead) and dead sperm (arrow) in an eosin smear (40X magnification).

Using an improved Neubauer hemocytometer, the sperm count (x10^6^ sperm/mL) was calculated in accordance with the^28^. To allow spermatozoa to exit the epididymal tubules, the two caudal epididymises were removed (Fig. 1A), cut with surgical scissors, and then incubated at 37 °C for 20 min. Next, via a Pasteur pipette, the sperm suspension was combined with 50 µl of semen and 950 µl of 40% formal saline (100 ml of formaldehyde, 9 g of sodium chloride, and 900 ml of distilled water) to dilute the semen sample 1:20.

The sperm count was performed according to the methods of the^28^; briefly, 10 µl of mixed diluted semen was loaded into each of the counting chambers of the improved Neubauer hemocytometer. A hemocytometer was placed on the microscope stage; a high-power objective (40X) was used to count the number of sperm heads (Fig. 1B). Two counts for each rat were recorded, and the mean sperm count was calculated as follows: mean sperm count = (count 1 + count 2)/2. The concentration of sperm per mL was determined according to the formula of^30^: the sperm concentration is equal to N × D × 10/A, where A is the area in square millimeters, D denotes the dilution factor, and N is the mean sperm count. The counting chamber has a 1 mm^2^ central area. Considering that the sperm count was conducted in five small squares, this area was divided into twenty-five smaller squares (1/25 mm^2^); hence, A = 1/5 = 0.2 mm^2^. The sperm concentration in mm^3^ was determined via this formula. The result was multiplied by 1000 to obtain the number in milliliters.

#### Sperm viability

The dye exclusion approach, which is based on the idea that dead cells with a broken plasma membrane permit membrane-impermanent stains, was used to identify sperm with an intact cell membrane to calculate the percentage of live spermatozoa. The dead spermatozoa heads are stained red or dark pink, but the heads of the live spermatozoa are white or light pink (Fig. 1C). Eosin staining was used to confirm viability in accordance with^28^. The average number of living and dead spermatozoa was calculated after nearly three replicates were evaluated, each containing 200 spermatozoa. The average live sperm/average live and dead sperm ×100 is the percentage of viability.

#### Analysis of sperm morphology

After semen collection, the samples were fixed with Hancock’s solution^31^. In accordance with the methods of the^28^, smears were prepared, and dried smears were stained with Giemsa for 10 min and then analyzed at 40X magnification^32^. The following definitions were considered during the classification process: headless is a spermatozoon without a head, or one where the head was completely separated; pin head is abnormally small or tapered heads, where the size of the head was less than 50% of the original size; broken head is structural damage to the head; bent neck is where the angle between the head and midpiece was greater than 90 degrees; broken tails are structural damage to the flagellum, including both principal and end pieces; tailless is a spermatozoon without a flagellum; coiled tails are flagellum coiled around the perimeter of the head; bent tails where there was a sharp angle greater than 90 degrees between two points along the flagellum; curved tails where there was a smooth curvature between two points along the flagellum. Each rat’s 200 spermatozoa were examined, and the number of normal and abnormal sperm was determined. By computing the average number of normal shapes to the closest whole number, x100, the average percentage of normal forms was determined. Head and tail abnormalities are two categories of morphological abnormalities. The average number of abnormal sperm equal to the total number ×100 was used to calculate the percentages of sperm with abnormal morphology.

### Statistical analysis

Statistical analysis was performed employing OBM SPSS version 30, with a significance level of *P* < .05. The normality of the data was checked using the Shapiro-Wilk normality test, which guided the use of parametric or non-parametric tests. Student’s t-test or one-way analysis of variance was used for normally distributed data, while the Mann-Whitney U test and chi-square test were used for the analysis of abnormally distributed sperm parameters. For ordinal and categorical data, such as lesion scores and clinical scores, the data were analyzed using the Pearson chi-square test with Monte Carlo simulation. The correlation between parasitology and semen data was analyzed using Spearman’s rank correlation test. The analysis was independent across animal groups, and no correction for multiple testing was made, which increased the risk of Type 1 errors. No adjustment for multiple comparisons was made regarding the results of the assessed variables over time. This is in accordance with the exploratory descriptive nature of the research. The risk of Type I error is increased, and results should be interpreted with caution for those that have a P value between 0.01 and 0.05.

## Results

A total of thirty-six male Wistar rats were included in this investigation. Twenty-four rats were orally infected with the Type II Me49 strain of *T. gondii* (15 tissue cysts/rat), whereas twelve rats were in the control group.

### Evaluation of acute toxoplasmosis

During the whole experimental period, all the control group rats were free of clinical signs of acute toxoplasmosis. They had normal posture, and their fur was glossy and silky. All the rats infected with *T. gondii* survived the acute phase of the infection, which was resolved three weeks after infection. All the rats exhibited no clinical signs of acute toxoplasmosis at week one PI (score 0). Only one rat was reluctant to move (score 2), and two of the twenty-four rats had a score of 1 at weeks two and three PI because of their hunched posture and ruffled fur. Over the course of the five weeks, there was negligible variation in the scores (*P*=.130). These clinical signs, however, disappeared after the third week PI, and the rats continued to be active until the end of the study. From week 2 to week 5 PI, the body weights of the rats infected with *T. gondii* were significantly lower than those of the control group (*P* < .05).

### Evaluation of chronic toxoplasmosis

#### Body weight during chronic infection

The body weights of *T. gondii* -infected rats were significantly lower at all designated time points (40, 50, and 60 days PI) *than* those of the control group (*P*< .05), as shown in Table 1.

**Table 1:** Body weight follow-up in the infected and control groups before euthanization at days 40, 50 and 60 postinfection.

Body weight/gm			*P*- value
Time points	Infected group	Control group	
Day 0	261.25 ± 14.0	261.58 ± 11.60	0.944
Day 40	270.88 ± 18.64 (12.3%*)	308.75 ± 13.84	< 0.001
Day 50	252.25 ± 11.23 (19.4%*)	313.5 ± 11.38	< 0.001
Day 60	260.88 ± 21.15 (19%*)	321.25 ± 15.48	< 0.001

The data are presented as the means ± SDs.

* Values in parentheses represent the percentage reduction in body weight of the infected group relative to the contemporaneous control group mean, calculated as [(control mean − infected mean)/ control mean] × 100.

Significant difference from the control group at *p* < .05. Test of significance: Student’s t-Test.

#### Assessment of the brain cyst burden

To confirm chronic toxoplasmosis in infected rats, the number of *T. gondii* cysts in brain homogenates was determined. *T. gondii* cysts were found in the brain tissue homogenate from every infected rat. According to Table 2, on day 40 PI, the mean tissue cyst burden was 1275; at 50 days PI, it was 1300; and at 60 days PI, it was 1350. The differences in cyst burden across the time points were statistically insignificant (*P*>.05).

**Table 2:** Brain cyst burden in Toxoplasma-infected male rats at 40, 50, and 60 days post infection.

Days postinfection	Cyst count in 10µ brain homogenate	Cyst burden
Day 40	6.375 ± 1.06	1275 ± 212.13
Day 50	6.5 ± 1.85	1300 ± 370.32
Day 60	6.75 ± 1.8	1350 ± 366.45
	*P*= .810	*P*= .812

Normally distributed data is expressed as the means ± SDs and were compared via the F test.

ANOVA.

#### Histopathological examination of brain tissue

**Fig. 2:**
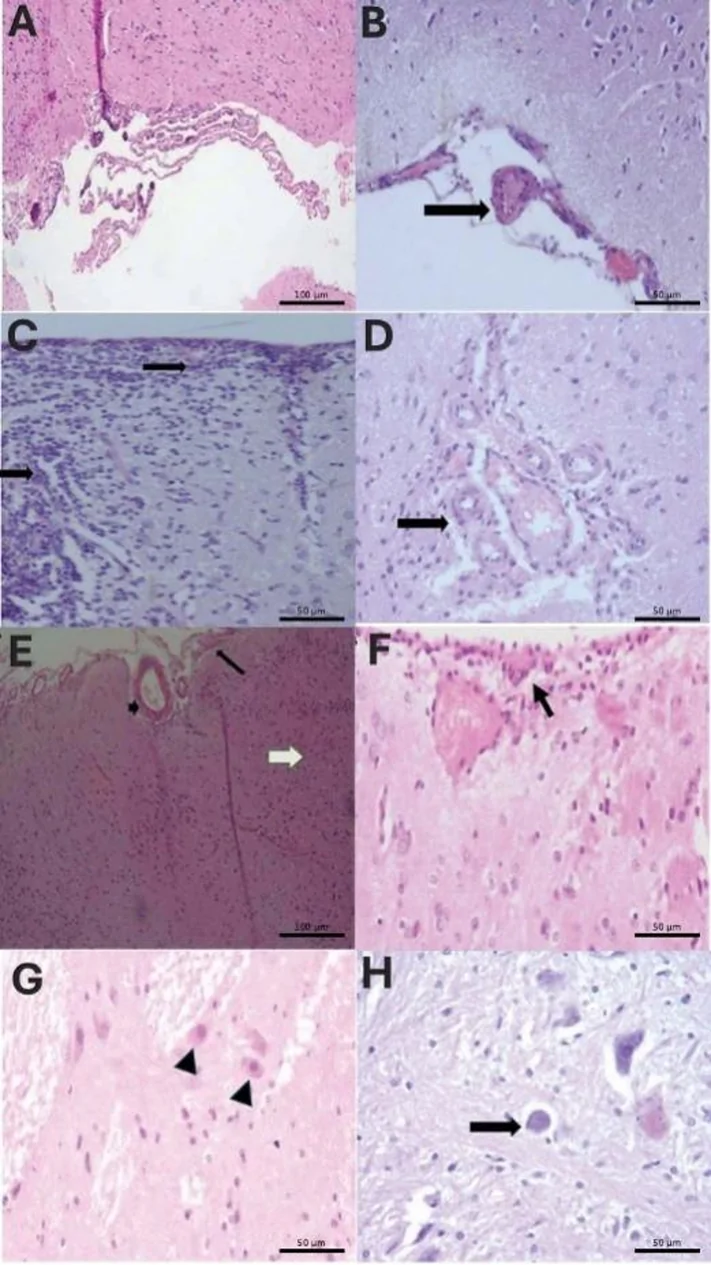
Cross-sections of H&E-stained brain tissue (**A**) from a control rat (Magnification, 200x) showing normal meninges and blood vessels with normal brain cellularity, (**B**) Brain tissue from a *Toxoplasma*-infected rat (at day 60 PI (Magnification, 400x), showing meningeal vascular congestion (black arrow), (**C**) Meningeal lymphocytic infiltration with perivascular lymphocytic infiltration (black arrows) (at day 60 PI) (Magnification, 400x), (**D**) Dilated congested blood vessels surrounded by lymphocytic infiltration (at day 60 PI) (Magnification, 400x), (**E**) Reactive meningitis (black arrow), congested blood vessels (arrowhead), and glial cell infiltration, (White arrow), representing inflammatory score II (at day 50 PI) (Magnification, 200x), (**F**) Reactive meningitis (black arrows) representing inflammatory score II (at day 50 PI) stained with H &E (Magnification, 400x), (**G**) Neuronal degeneration of the nerve cell body (arrows), with focal neuropil rarefaction (Inflammatory score III) (at day 40 PI) (Magnification, 400x), and (**H**) *Toxoplasma* cyst (arrow) (Magnification, 400x).

Histopathological examination of the control rat brain tissue revealed no inflammation of the brain parenchyma, normal meninges, or non-congested blood vessels, as shown in Fig. 2A. Inflammation of the meninges, blood vessel congestion, and inflammation of the brain parenchyma or encephalitis were observed in the *T. gondii*-infected rat group brain tissue (Fig. 2B and C). According to Table 3, the scoring of histopathological lesions revealed that nearly all lesions at 50- and 60-days PI were mild (score II), indicating meningitis, perivascular cuffs, and local glial cell infiltration, as shown in Fig. 2D, E and F. As shown in Fig. 2G, most lesions at 40 days PI were moderate (scoring III), including meningitis, perivascular cuffs, glial cell infiltration, and focal neuropil rarefaction. As shown in Fig. 2H, T. *gondii* tissue cysts were discovered in the infected group.

**Table 3:** Scoring of histopathological lesions in brain tissue of *T. gondii-*infected rats.

Days postinfection	Number of rats	Scoring of brain lesions				
		0	I	II	III	IV
Day 40	8	-	1 (12.5%)	2 (25%)	5 (62.5%)	-
Day 50	8	-	6 (75.0%)	1 (12.5%)	1 (12.5%)	-
Day 60	8	-	4 (50%)	2 (12.5%)	2 (12.5%)	-

Monte Carlo, *P* = .130.

The values are expressed as the number and percentage of rats.

### Fertility indices assessment

#### Body weight to testis weight ratio

The mean testicular weights of the infected and uninfected groups were determined. Compared with control rats, male rats infected with *T. gondii* had significantly lower mean testicular weights at 40, 50, and 60 days PI (*P*< .05). The decline was more pronounced at 60 days PI (*P*< .01).

#### Sperm parameter analysis

##### Sperm motility

The median percentage of progressively motile sperms was significantly decreased in the *T. gondii* -infected group when compared to the controls on day 40 PI (*P* = .01) and day 50 PI (*P* = .049). The decrease in the median percentage of progressively motile sperms on day 60 PI was more significant (*P* = .008), as depicted in Table 4.

**Table 4:** Sperm motility, count, viability, and morphology in *Toxoplasma*-infected and control male rats.

Days postinfection	Infected group	Control group	*P*- value
Sperm motility %			
Day 40	30 (11–56) *	58.5(54.3–65)	0.01
Day 50	32 (14–50) *	53.5(49–60)	0.049
Day 60	37 (9–45) **	53.75(52–57)	0.008
Sperm count (x10^6^/ml)			
Day 40	20 (6–34) **	51(36–56)	0.006
Day 50	21 (0.5–32) **	47(37–56)	0.007
Day 60	22 (10–40) **	46.5(42–60)	0.007
Sperm viability %			
Day 40	61 (52-86.5) *	83.7(80-85.4)	0.04
Day 50	69 (48–70) *	81.25(78–85)	0.03
Day 60	70 (57–88) *	82.65(79–86)	0.04
Normal sperm morphology %			
Day 40	48.75 (27.5–65.5) *	76.5 (72.5–79)	0.007
Day 50	57.25 (32–70) *	77.75 (76–81)	0.007
Day 60	58 (37–64) *	79.25 (76–80)	0.007

Data is presented as the median (range). *Significant difference from the control group at *P* < .05, **Significant difference from the control group at *P*<.01, Test of significance: Mann‒Whitney U test, chi-square test.

##### Sperm count

At all designated time intervals of infection (40, 50, and 60 days PI), the median sperm count in the infected rat group was significantly lower than that in the control group, as shown in the statistical analysis of the obtained results (*P*<.05) in Table 4.

##### Sperm viability

Table 4 shows the findings of the assessment of rat sperm viability in the study groups. At 40, 50, and 60 days PI, the median percentage of viable sperm in the *T. gondii* -infected group was considerably lower than that in the control group (*P* < .05).

##### Sperm morphology

The percentage of normal rat sperm in the infected rat group varied from 48.75% to 58%, whereas in the control group, it ranged from 76.5% to 79.25%. The lower percentage of normal sperm in the *T. gondii* -infected rats was statistically significant at every time point (Table 4). According to the statistical analysis of the data, the *T. gondii*-infected rat group presented a significantly greater median percentage of total sperm morphological abnormalities (teratosperms) at all time points than did the control group (*P*<.05). With regard to individual rat sperm abnormalities, as shown in Table 5, on day 40 PI, the median proportion of headless and tailless sperm deformities was considerably greater in the infected rats than in the control rats (*P*<.05).

**Table 5:** Total and individual sperm abnormalities in *Toxoplasma*-infected and control male rats at days 40, 50, and 60 days postinfection.

	Infected group	Control group	*P*- value
**On day 40 postinfection**			
Teratosperms (%)	51.25 (34.5–72.5)**	23.25 (21.5–27.5)	0.006
Head abnormality			
Head less (%)	8 (5–17)**	3.25 (2-3.5)	0.006
Pin head (%)	0.5 (0-1.5)	0 (0-0.5)	0.343
Broken head (%)	2.0 (0.5–6)	0.5 (0.5 − 1.0)	0.06
Neck abnormality			
Bent neck (%)	1.5 (1–3)	1 (0–3.0)	0.833
Tail abnormality			
Broken tail (%)	0.5(0–8)	0.25 (0-0.5)	0.297
Tail less (%)	12.75 (6.5–19)*	6.25 (5–8)	0.013
Coiled tail (%)	4.25 (0.5–6)	3 (2-3.5)	0.443
Bent tail	15 (7–27)	7.5 (5-9.5)	0.06
Curved tail	2.5 (1–5)	2.25 (1–3)	0.602
**On day 50 postinfection**			
Teratosperms (%)	42.75 (30–68)**	22.25 (19–24)	0.007
Head abnormality			
Head less (%)	10.5 (6–18)**	5.25 (3.5-6)	0.008
Pin head (%)	0.75 (0–1.0)	0.5 (0-0.5)	0.074
Broken head (%)	1.75 (1-2.5)	0.25 (0–1)	0.076
Neck abnormality			
Bent neck (%)	1.5 (0.5-4.5)	0.75 (0.5 − 1.0)	0.158
Tail abnormality			
Broken tail (%)	2.5 (1–10)	1.5 (0.5-2.5)	0.145
Tail less (%)	7.75 (3-24.5)*	3.5 (3–5)	0.03
Coiled tail (%)	3.75 (1–5)	1.25 (0.5-2.5)	0.073
Bent tail	12 (9–15)**	6.75 (6–8)	0.006
Curved tail	2.25 (0.5-4.0)	2 (0.5 − 3.0)	0.794
**On day 60 postinfection**			
Teratosperms (%)	42 (36–63)**	20.75 (20–24)	0.007
Head abnormality			
Head less (%)	6.75 (2.5–26.5)	3.75 (3–6)	0.074
Pin head (%)	1.0 (0.5 − 3.0)	0 (0-0.5)	0.346
Broken head (%)	1.25 (0.5 − 3.0)	0.75 (0.5 − 1.0)	0.158
Neck abnormality			
Bent neck (%)	1.25 (0–3.0)	0.75 (0.5 − 1.0)	0.171
Tail abnormality			
Broken tail (%)	2 (0.5 − 4.0)	1 (0.5-1.0)	0.062
Tail less (%)	9.25 (3.5–16)*	4.25 (3.5-6)	0.048
Coiled tail (%)	3.75 (2–7)*	2 (1.5–2.5)	0.016
Bent tail	12.75 (7–16)**	6 (4–7)	0.008
Curved tail	4 (3–5)**	2.5 (2.5-3)	0.009

Data is presented as the median (range). *Significant difference from the control group at *P*<.05. **Significant difference from the control group at *P*<.01. Test of significance: Mann‒Whitney U test, chi-square test.

**Fig. 3:**
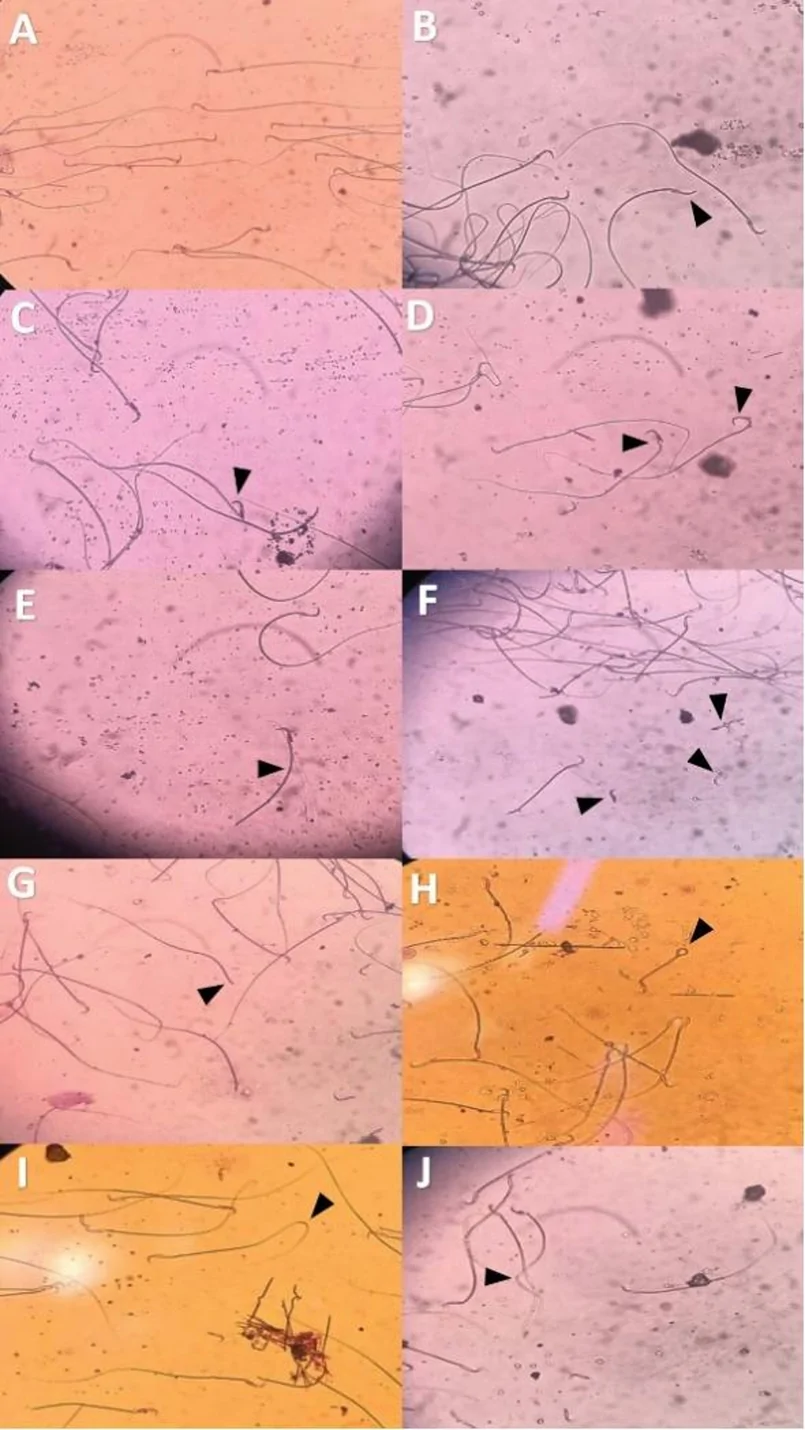
(**A**) Normal sperm and morphological changes (arrowhead) in the sperm of the *T. gondii*-infected rat group (40X magnification). (**B**) Pin head sperm, (**C**) sperm with a broken head, (**D**) sperm with a bent neck, (**E**) rat sperm with a broken tail, (**F**) tailless rat sperm, (**G**) headless rat sperm, (**H**) rat sperm with a coiled tail, (**I**) rat sperm with a bent tail, and (**J**) rat sperm with a curved tail.

At 50 days PI, the median percentage of teratosperms, including those with no head, bent tail or without tail, was significantly greater in the *T. gondii* -infected group than in the control group (*P* < .05), as presented in Table 5. At 60-days PI, the infected group presented considerably more sperm tail abnormalities than the control group did, including sperm with no tail, a coiled tail, a curved tail, and a bent tail (*P*<.05). Figure 3A shows normal rat sperm, while Fig. 3B and J show an increase in various rat teratosperms in the *T. gondii* -infected group.

#### Correlations between sperm parameters and brain lesions

Spearman’s rank correlation test results showed no statistically significant correlation between the parasite load of the brain and any of the tested semen parameters such as percentage of viable sperm, motile sperm, total sperm count, normal morphologic forms, and total number of teratosperms. Likewise, there was no statistically significant correlation between the score for brain inflammation and any semen parameter (Table 6).

**Table 6:** Correlations between sperm parameters and brain lesions.

		Viability	Motility	sperm count	Normal morphology	Teratosperms
Brain cyst burden	R	− 0.271	− 0.083	− 0.056	− 0.140	0.140
	*P*	0.201	0.720	0.796	0.513	0.513
Score of brain inflammation	R	0.341	0.292	− 0.161	− 0.042	0.042
	*P*	0.103	0.199	0.452	0.845	0.845

R: correlation, P: P value, * significant correlation at *P* < .05, ** double significant correlation at *P* < .01.

## Discussion

With a wide host range, *T. gondii* is an obligatory intracellular protozoan parasite. Although *T. gondii* infections are prevalent worldwide, toxoplasmosis is regarded as a tropical neglected disease. and is often disregarded in reproductive health clinics in various countries^33^. Male idiopathic infertility may be caused by toxoplasmosis, which has a significant and potentially detrimental effect on the male reproductive system. Consequently, the aim of this study was to investigate how chronic toxoplasmosis affects the semen parameters of male rat which is an indicator of the rat potential fertility.

Rats are considered a good model for studies on human toxoplasmosis. Compared with mice, rats more closely resemble human response to toxoplasmosis due to their greater resilience to clinical disease^20^. This study examined the impacts of chronic toxoplasmosis on male reproductive indices, particularly semen, via an experimental male Wistar rat model of the disease and the type II Me49 strain of *T. gondii*. The first and most important step in evaluating male fertility potential in the laboratory is semen analysis. Male rat semen parameters were investigated in this study as key indicators of reduced fertility potential.

In this study, the *Toxoplasma*-infected rats exhibited mild, temporary clinical signs of acute toxoplasmosis. These clinical signs eventually disappeared until the end of the experiment (60 days PI). Male rats typically show no clinical signs of acute toxoplasmosis^24^. In contrast, during the acute phase of toxoplasmosis^34] and [35^, reported behavioral changes with less illness in rats infected with the *T. gondii* less virulent type II strain. However, mice infected with a virulent strain of *T. gondii* type I suffer more severe illness^36^. Accordingly, the behavioral outcome of *T. gondii* infection is significantly influenced by the host–parasite strain interaction^37^.

Based on our findings, rats infected with *T. gondii* lost a substantial amount of body weight compared with the control group. These findings support the results of Taherimoghaddam et al.^38^, who reported that *T. gondii* infection in male Wistar rats was associated with a notable decrease in body weight by 20 days PI, which indicates the acute stage of toxoplasmosis. Compared with mice that were not infected, parasite invasion was associated with a statistically significant reduction in body weight three weeks after infection, as well as a progressive loss in body weight over the course of the illness^39^. Furthermore, outbred mice inoculated subcutaneously with *T. gondii* tissue cysts survived and experienced modest sickness in the form of weight loss in the second and third weeks after infection, according to Dubey^24^.

The results of the present study revealed that, starting on day 40 PI, which correlated with the chronic phase of toxoplasmosis, and continuing through days 50 and 60 PI, the body weight of the *Toxoplasma*-infected group was significantly lower than that of the control group. Similarly, previous study reported that after losing up to 20% of their initial body mass in the first three weeks following infection, adult mice that were orally infected with ten tissue cysts of the Type II Me49 strain *T. gondii* suffered significant wasting that lasted for up to 21 weeks PI^40^. The same study revealed that mice with chronic toxoplasmosis presented increased levels of the cachexia cytokines IL-1β, TNF-α, IFN-γ and IL-6. Baazim et al.^41^ reported that IL-1 contributes to the breakdown of muscle and adipose tissues. Compared with that of the control group, the mean testicular weight of the *T. gondii*-infected rats was significantly lower at all time points. In line with these findings, male rats infected with *T. gondii* presented a notable decrease in testicular weight at 30 days PI^42^ and at 60 days PI^43^ compared with the uninfected group. The reduction in body weight seen in the infected rats may affect their semen parameters by virtue of the systemic metabolic effect of reduced nutrition and cachexia associated with infection. This experiment did not make use of pair-fed controls and thus there was no means of accounting for the systemic metabolic effects associated with infection-induced weight loss and its impact on the observed reduction in semen parameters. Hence it is impossible, based on these results, to distinguish between effects of *T. gondii* infection specific to reproductive organs and effects associated with the systemic metabolic impact of infection-induced weight loss. The use of various *T. gondii* strains may be the cause of this discrepancy in outcomes. Potential sources of inter-study variability in reproductive consequences of *T. gondii* infection include but are not limited to parasite genotype. The mode of parasite inoculation determines the immune response characteristics of the host: orally administered infection initiates a specific mucosal immunity via TLR-mediated pathways and induces Th1 response, while intraperitoneal inoculation circumvents the intestine epithelium and results in accelerated systemic tachyzoite spread^44^. Large inoculum concentrations could enhance parasite proliferation during the initial stage of infection and result in enhanced infection spreading before cysts formation, which in turn leads to greater chances of infection of the gonads^24^. Differences in hosts’ species/strain characteristics can be another contributing factor to variability: sensitivity to persistent infection, permeability of blood-testis barrier, and establishment of testicular immune privilege differ significantly between various rodent strains^45^. Thirdly, the timing of reproduction evaluation is paramount because research that terminates experiments after acute-to-chronic transition phase (Days 10–40 PI) differs from research conducted after chronic infection phase (≥ 60 days PI)^12^. The need for standardization of these variables is vital for any inter-comparison between research studies.

According to our results, toxoplasmosis in rats had negative effects on sperm count, viability, motility, and morphology for up to 60 days PI which is consistency with other studies^13,46^. Other studies revealed that during the acute phase of toxoplasmosis, male rats infected with *T. gondii* presented an increase in the overall number of sperm abnormalities and a decrease in all semen parameters, including sperm count, sperm motility, and sperm viability^38^. Chabuk^47^ reported that, from 6 weeks to 12 weeks after *T. gondii* infection, the sperm parameters of male rats were affected. Similar results were also observed in male mice given a 10-day PI dose of *T. gondii*. While sperm motility remained unchanged, male mice treated with *T. gondii* 28 days after infection also presented a decreased total sperm count and an increased rate of sperm abnormalities^48^.

Few studies on how human seminal parameters are affected by chronic toxoplasmosis. In a pilot study including 60 males (15 of whom tested positive for *T. gondii* ), no significant effects of *T. gondii* infection on sperm characteristics, specifically sperm count, motility, and morphology, may be attributed to the limited sample size^49^. Latent toxoplasmosis, however, was found to influence sperm motility and count in a cross-sectional investigation of 669 males but not on sperm morphology or semen volume. Men with abnormal semen standards, such as low sperm count, low motility, and a low percentage of normal sperm, have been reported to have a higher prevalence of latent toxoplasmosis than men with normal semen characteristics^50^.

The present study clearly indicates the impaired semen quality in chronically infected male Wistar rats; however, the mechanism of the effect of T. gondii remains beyond the scope of the present study. No endocrine, immunological, or oxidative stress-related factors were evaluated in the present study, and the mechanistic explanations should be considered with reference to literature. Firstly, although tachyzoite invasion of the testis has been demonstrated in earlier experimental studies^11,12^, reproductive tissues were not examined in the present work; the tissue-level basis of the semen abnormalities observed here therefore remains undetermined and any direct testicular or epididymal injury is proposed only as a possible mechanism based on the literature. Secondly, the oxidative stress caused by the infection, which is supported by increased levels of malondialdehyde and decreased superoxide dismutase activity in the testes of acutely infected rodents^13,16^, could impair the process of spermatogenesis directly through the oxidative modification of the plasma membrane and DNA of the sperm cells^17,51^. The hypothalamic-pituitary-gonadal axis abnormalities were demonstrated in *T. gondii*-infected rodents, which could be the mechanism of the effect of the parasites on the process of spermatogenesis through the reduction of serum testosterone^52^. Fourth, immune-mediated pathways, such as elevated anti-sperm antibody levels in *T. gondii* seropositive infertile men^7^, may also play a role in sperm agglutination and dysfunction. Whether or not any of these pathways are responsible for the abnormalities in semen parameters found in this study is unknown and represents an area of future research.

According to the results obtained in the current experiment, there were no significant associations found among brain parasitological variables (either brain cyst burden or brain inflammation score) and seminal characteristics using Spearman’s rank correlation. However, these data must be cautiously interpreted due to low statistical power associated with small number of subjects included (*n* = 8 per each group) to ensure sufficient data variability at each time point. Furthermore, it is also necessary to emphasize that brain cyst burden may not necessarily reflect local infection or inflammation in testes and epididymides, whose participation in pathogenesis of chronic toxoplasmosis was not studied in the current experiment.

A limitation of this current study is the lack of formal priori power calculations to determine the power of this study to detect changes in each of the endpoints examined. The sample sizes utilized in this study, although consistent with the experimental toxoplasmosis literature, may have been inadequate to detect subtle changes or to provide sufficient power to detect changes in the secondary endpoints of individual sperm morphological subtypes. Future studies must include power calculations to determine the power of this study to detect changes in all endpoints of interest. Another limitation is that testes and epididymides were not histologically evaluated, which made it impossible to elucidate the morphological background of the abnormalities in the semen parameters. Histopathological examination of the seminiferous tubules and the epithelium of the epididymis would greatly enhance the results obtained from the semen analysis.

## Conclusion

This study provides compelling evidence that chronic toxoplasmosis significantly impairs semen quality in experimentally infected male rats. Infection was associated with significant reduction in sperm count, motility, and viability, as well as a marked increase in morphological abnormal spermatozoa. The findings obtained in this investigation, in an experimental animal model, support the hypothesis that chronic *T. gondii* infection may be a contributing factor in diminished male reproductive potential; however, future studies are crucial to investigate the precise mechanisms driving these reproductive changes and to evaluate the relevance of these findings in the human population.

## Data Availability

The datasets used and/or analysed during the current study are available from the corresponding author (NE) on reasonable request.
